# Severe late-onset multisystem cytomegalovirus infection in a premature neonate previously treated for congenital infection

**DOI:** 10.1186/1471-2431-13-142

**Published:** 2013-09-11

**Authors:** Manal F El-Sayed, David M Goldfarb, Martha Fulford, Jeffrey M Pernica

**Affiliations:** 1 Department of Pediatrics, McMaster University, Hamilton ON, Canada; 2 Division of Infectious Disease, Department of Pediatrics, McMaster University, rm 3A-30 1280 Main St. West, Hamilton, ON L8S 4 K1, Canada; 3 Division of Infectious Disease, Department of Medicine, McMaster University, Hamilton, ON Canada

**Keywords:** Cytomegalovirus, Congenital infection, Ganciclovir

## Abstract

**Background:**

Cytomegalovirus is the most common pathogen causing congenital infection and can result in significant neurodevelopmental adverse outcomes. For this reason, it is the standard of care in many regions to treat congenital cytomegalovirus infection involving the brain with six weeks of ganciclovir. There have been no reports in the published literature of significant cytomegalovirus neonatal infection in infants previously treated for congenital infection.

**Case presentation:**

A preterm male infant with congenital symptomatic cytomegalovirus infection was initially treated with over 8 weeks of ganciclovir between the ages of 3 and 14 weeks. At four months chronologic age, just prior to planned discharge, he developed an episode of life-threatening multisystem cytomegalovirus disease notable for severe pneumonitis, encephalitis, hepatitis, and disseminated intravascular coagulation. This disease resolved after re-treatment with a prolonged course of intravenous ganciclovir and oral valganciclovir.

**Conclusions:**

Clinicians should be aware of the possibility of recurrence of congenital cytomegalovirus infection, especially in preterm infants. Serial plasma cytomegalovirus viral load monitoring may have a role in the management of premature infants treated with ganciclovir; had the diagnosis of recrudescent cytomegalovirus infection been considered sooner, specific therapy might have been more quickly initiated and perhaps further morbidity would have been prevented.

## Background

Cytomegalovirus (CMV) is currently the most common pathogen causing congenital (fetal) infection. Sequelae of congenital infection include significant cognitive deficits, psychomotor impairment and deafness. As a result, it is now the standard of care in many regions to treat congenital CMV infection (especially that involving the central nervous system) with at least six weeks of ganciclovir in an effort to minimize neurodevelopmental sequelae, given the decrease in hearing loss seen with this therapy in a randomized controlled trial [1].

CMV infection can also be acquired in the perinatal or postnatal periods through contact with genital secretions, horizontal close contact, blood product transfusions, or via ingestion of breast milk contaminated with the virus. Perinatally- or postnatally-acquired CMV infection, in contrast to congenital infection, more frequently presents with a ‘sepsis-like syndrome’ or severe pneumonitis [2]; numerous case reports document severe illness caused by postnatally-acquired CMV in very low birth weight infants [3,4].

## Case presentation

The patient was a 730 g male born at 28^+1^ weeks of gestation, the product of a spontaneous dichorionic diamniotic twin pregnancy complicated by severe growth restriction in both fetuses. Leukopenia, thrombocytopenia, and mild bilateral ventriculomegaly were present at birth, which raised suspicion for congenital infection; a urine sample sent on day 12 of life was positive for CMV by culture. Blood work demonstrated very mildly elevated serum conjugated bilirubin (13 micromol/L) on the day of birth that progressively increased; there was no elevation of liver transaminases, increase in prothrombin time, or clinically evident bleeding. CMV infection of both placentas was confirmed using immunohistochemistry and both chronic hemorrhagic lymphoplasmacytic villitis and villous vasculitis was seen on pathology. Consequently, treatment with ganciclovir (6 mg/kg/dose IV q12h) was initiated at 19 days of age. It should be noted that his absolute neutrophil count (ANC) transiently dropped below 1000/microL at 17 days of age, *before* antivirals were started. On day 2 of ganciclovir treatment, the ANC decreased again to 800/microL, which resulted in a 36-hour suspension of antiviral therapy.

At just under 5 weeks chronologic age, after 13 days of ganciclovir therapy, when his absolute neutrophil count was normal, the patient developed sepsis in association with an *Enterococcus faecalis* central line infection. The peripherally-inserted central catheter was not removed promptly, his neutrophil count briefly dropped to 400/microL, the ganciclovir was held, and he experienced a pulmonary haemorrhage four days later. With antibacterial therapy and removal of the offending venous catheter, his condition rapidly stabilized. His plasma CMV viral load (VL) was determined to be 1 × 10^6^ copies/mL after eleven days off treatment; ganciclovir treatment was resumed after a two-week hiatus and continued for an additional 6 weeks through a replacement central venous catheter. Due to the neutropaenia associated with his septic deterioration, filgrastim (granulocyte colony stimulating factor) therapy was begun the day prior to the reinitiation of ganciclovir therapy and was given intermittently whenever the patient’s neutrophils appeared to be in decline, though the ANC did not go below 1000/microL during the remainder of the ganciclovir treatment course.

During this time, the baby was mainly fed with frozen-thawed breast milk. For prematurity-associated anaemia, he was transfused a total of 785 mL (4 donors) of leukoreduced irradiated blood, of which only 341 mL (2 donors) was received when not on ganciclovir therapy.

Three weeks after finishing the ganciclovir, at 4 months chronologic age, shortly before the time of his planned hospital discharge, the infant developed progressive respiratory distress. Cefazolin and gentamicin were started; blood cultures were negative, and, at this time, the absolute neutrophil count was normal. He was stable enough to be electively intubated for laser eye surgery, but subsequently developed increased respiratory requirements. His chest X-ray showed bilateral mixed airspace and interstitial opacities, an endotracheal tube aspirate showed ‘few’ WBCs on the gram stain, and aspirate cultures grew *K. pneumoniae* (sensitive to cefazolin), so a presumptive diagnosis of ventilator-associated pneumonia was made. The cerebrospinal fluid was sampled and was found to have 13 WBC/hpf (2% neutrophils, 48% lymphocytes, 50% monocytes), 2 RBC/hpf, glucose of 2.8 mmol/L, and protein of 1.3 g/L. His condition worsened, and he was switched to cefotaxime, without demonstrable improvement. He developed respiratory failure requiring ventilation with high frequency oscillatory ventilation and nitric oxide therapy was initiated. Significant hepatosplenomegaly was observed for the first time, accompanied by elevated AST and ALT (273 U/L and 221 U/L, respectively), and the patient developed disseminated intravascular coagulation. Repeat bacterial cultures of blood, urine and cerebrospinal fluid were negative. Given the pneumonitis, massive hepatosplenomegaly, thrombocytopenia, coagulopathy, and lack of response to cefotaxime, CMV was suspected to be the causative pathogen. Urine and endotracheal aspirate cultures for CMV were positive, plasma CMV polymerase chain reaction (PCR) showed a VL of 3 × 10^6^ copies/ml, and CMV was detected in the CSF by PCR. Cefotaxime was discontinued and ganciclovir therapy was reinitiated (6 mg/kg/dose IV q12h) 14 days after the onset of his deterioration; slow improvements in respiratory status and thrombocytopenia were then observed. After 13 days of therapy, the infant was stable from a cardiopulmonary perspective; his CMV plasma VL at that time was 1865 copies/mL. The patient continued to improve and after 4 weeks of IV therapy he was switched to oral valganciclovir (16 mg/kg/dose q12h) when he no longer required intravenous access for any other indication.

He was discharged home three weeks later, on valganciclovir, at 6 months of chronologic age, still requiring low flow oxygen; at this time, his CMV plasma VL was 5771 copies/mL. The decision was made to continue oral antiviral therapy after discharge until his viral load became undetectable, given his complicated history; ultimately, he received 9 weeks of oral therapy and ~ 3 months total therapy. He did not experience any significant complications thought to be caused by his second treatment course of ganciclovir/valganciclovir; his absolute neutrophil count hit a nadir of 900/microL but recovered without any filgrastim treatment or alteration to antiviral dosing. Laboratory evaluation for immune deficiency performed at 11 months chronologic age demonstrated that his immunoglobulin A level was 0.31 g/L, immunoglobulin G level was 14.6 g/L, and immunoglobulin M level was 0.95 g/L. The patient’s CD4 count was 1530/microL (30%), CD8 count was 1580/microL, and he had 23% CD19 B cells. Phytohemagglutinin stimulation testing of T lymphocytes revealed a normal response.

## Conclusions

The clinical presentation and sequelae associated with congenital CMV disease have been described in detail; however, this is the first reported case of which we are aware to describe an infant treated for congenital CMV infection who later in infancy had a life-threatening episode of presumed multi-organ CMV infection. We hypothesize the following: first, that the episode of life-threatening illness that the patient experienced at four months chronologic age was caused by CMV, and second, that this CMV infection was due to reactivation of congenitally-acquired virus rather than to a second, postnatally-acquired, infection.

We posit that CMV infection was responsible for the infant’s clinical deterioration at four months of age because of the clinical picture of pneumonitis, hepatitis, hepatosplenomegaly, thrombocytopenia, and coagulopathy; furthermore, the patient progressively deteriorated while on antibacterials, had a greatly elevated CMV plasma VL, and had CMV detected in all fluids assayed with zero sterile-site cultures positive for a bacterial pathogen. Other aetiologies cannot be definitively excluded, however, as herpesviruses can reactivate leading to DNAemia in the context of an intercurrent infection. The CMV VL at the time of his late clinical deterioration was not significantly different than the VL after his first course of ganciclovir was prematurely interrupted, though it was clearly higher than all the VLs recorded subsequent to his re-treatment with ganciclovir. The patterns of plasma CMV PCR VLs in infants with congenital infection subsequent to termination of therapy have not been systematically studied, though Kimberlin et al. documented a ~1 log increase in CMV VL in a small group of infants two weeks after the discontinuation of 6 weeks of ganciclovir therapy [5]; we note that CMV plasma VLs in the millions are not commonly found in ‘asymptomatic’ term neonates with congenital CMV infection [6].

If the episode of clinical deterioration at four months of age was indeed caused by CMV, what was the pathogenesis of infection? There are two main possibilities: 1) recrudescence of congenitally-acquired virus, or 2) acquisition of a second, postnatal, infection. We posit that the first possibility is most likely; however, neither viral typing nor sequencing were done. Frozen-thawed breast milk carries a low risk of postnatal CMV transmission [7]; he also received few transfusions and was given only leukoreduced irradiated blood, so his risk of transfusion-associated CMV infection can also be estimated to be extremely low [8]. As for the pathogenesis of recrudescence of congenital infection, there are multiple possibilities: viral reactivation, lack of initial response due to interruption of therapy, ganciclovir-resistant virus, among others. The first – viral reactivation – would appear to be most likely. There is no evidence for ganciclovir resistance, given his clinical and virologic improvement while on ganciclovir. His initial treatment course was certainly not ‘standard’, but he received over 8 weeks of appropriately-dosed IV ganciclovir between the ages of 3 and 14 weeks, which we judged to be sufficient at the time.

Our case has potential implications for both diagnostic testing and patient follow-up; had the diagnosis of recrudescent CMV infection been considered sooner, we could have more quickly initiated specific therapy and perhaps reduced morbidity. To this end, it may be useful to monitor plasma CMV VL at cessation of therapy, 2–4 weeks later (to catch initial VL rebound), and then at the time of any clinical deterioration/systemic illness of uncertain aetiology, especially for babies not born at term.

This case also raises the question once again about the appropriate duration of therapy for congenital CMV infection. Some authors have reported their experiences with prolonging therapy in infants with congenital CMV infection [9,10]. As stated by Kimberlin et al., “…[rebound of VL post-discontinuation of ganciclovir] provides a rationale for the study of a treatment period longer than the currently-used 6-week period” [5]. We extended antiviral therapy beyond 6 weeks to treat our patient’s CMV recrudescence because of a possible ‘failure’ of the initial 6–9 week course of ganciclovir, the severity of his illness, and an ongoing detectable CMV VL. An ongoing multicentre trial comparing 6 weeks to 6 months of oral valganciclovir will hopefully help define the benefits and risks of longer treatment of symptomatic congenital CMV infection (NCT00466817, http://www.clinicaltrials.gov). It must be emphasized that treatment of congenital CMV infection often requires assiduous monitoring of clinical, biochemical, and virologic indices, and therefore cannot be easily protocolized.

## Consent

Written informed consent was obtained from the parent of this patient for publication of this Case Report. A copy of the written consent is available for review by the Editor of this journal.

## Abbreviations

CMV: Cytomegalovirus; VL: Viral load; PCR: Polymerase chain reaction.

## Competing interests

The authors declare that they have no competing interests.

## Authors’ contributions

MFE-S drafted the manuscript and did most of the literature review. DMG and MF made important contributions in the revision of the manuscript. JMP provided the outline of the paper, aided with the first draft, helped with the literature review, and was ultimately responsible for all revisions. All authors gave final approval for the version to be published.

## Pre-publication history

The pre-publication history for this paper can be accessed here:

http://www.biomedcentral.com/1471-2431/13/142/prepub
